# Continuous quality improvement in nephrology: a systematic review

**DOI:** 10.1186/s12882-016-0389-1

**Published:** 2016-11-24

**Authors:** Julie Wright Nunes, F. Jacob Seagull, Panduranga Rao, Jonathan H. Segal, Nandita S. Mani, Michael Heung

**Affiliations:** 1 Department of Internal Medicine, Division of Nephrology, University of Michigan Health System, Ann Arbor, MI USA; 2 Department of Learning Health Sciences, University of Michigan Medical School, Ann Arbor, MI USA; 3 Taubman Health Sciences Library, University of Michigan, Ann Arbor, MI USA

**Keywords:** Kidney disease, Continuous quality improvement, Systematic review

## Abstract

**Background:**

Continuous quality improvement (CQI) has been successfully applied in business and engineering for over 60 years. While using CQI techniques within nephrology has received increased attention, little is known about where, and with what measure of success, CQI can be attributed to improving outcomes within nephrology care. This is particularly important as payors’ focus on value-based healthcare and reimbursement is tied to achieving quality improvement thresholds. We conducted a systematic review of CQI applications in nephrology.

**Methods:**

Studies were identified from PubMed, MEDLINE, Scopus, Web of Science, CINAHL, Google Scholar, ProQuest Dissertation Abstracts and sources of grey literature (i.e., available in print/electronic format but not controlled by commercial publishers) between January 1, 2004 and October 13, 2014. We developed a systematic evaluation protocol and pre-defined criteria for review. All citations were reviewed by two reviewers with disagreements resolved by consensus.

**Results:**

We initially identified 468 publications; 40 were excluded as duplicates or not available/not in English. An additional 352 did not meet criteria for full review due to: 1. Not meeting criteria for inclusion = 196 (e.g., reviews, news articles, editorials) 2. Not nephrology-specific = 153, 3. Only available as abstracts = 3. Of 76 publications meeting criteria for full review, the majority [45 (61%)] focused on ESRD care. 74% explicitly stated use of specific CQI tools in their methods. The highest number of publications in a given year occurred in 2011 with 12 (16%) articles. 89% of studies were found in biomedical and allied health journals and most studies were performed in North America (52%). Only one was randomized and controlled although not blinded.

**Conclusions:**

Despite calls for healthcare reform and funding to inspire innovative research, we found few high quality studies either rigorously evaluating the use of CQI in nephrology or reporting best practices. More rigorous research is needed to assess the mechanisms and attributes by which CQI impacts outcomes before there is further promotion of its use for improvement and reimbursement purposes.

**Electronic supplementary material:**

The online version of this article (doi:10.1186/s12882-016-0389-1) contains supplementary material, which is available to authorized users.

## Background

The United States health care system is one of the most expensive in the world, yet it ranks lowest among developed countries on many aspects of quality and performance [1–3]. Projections indicate there will be an epidemic growth of many chronic diseases in the future, including chronic kidney disease (CKD) [4]. Thus, identifying and addressing barriers to the delivery of high-quality, low-cost care is of paramount importance.

Continuous quality improvement (CQI) provides a systematic way to identify and address barriers to better outcomes. There is no single definition of CQI, but generally it is a management technique and set of tools to optimize systems and continually improve processes [5, 6] (Table 1). CQI methods have been used in the business and engineering sectors since the 1940’s and adoption of these techniques preceded Japan’s rise as a leading manufacturer post World War II [7].

**Table 1:** Continuous Quality Improvement – Attributes and Potential Associated Activities—adapted from Juran’s Quality Handbook [6]

Attributes of continuous quality improvement	Example(s) of associated activities
Highly team based and multi-disciplinary	Bringing together staff from management, administration, and ‘front-lines’ to meet for improvement goal
Understanding a process-either to identify areas of improvement or better understand how things get done	Process mapping
	Flow diagrams
Assessing current capabilities of process or system	Control charts
	Statistical process control
Generation and arranging of theories for why problems exist	Tabulation methods
	Cause/effect (Ishikawa) diagrams
Process dissection, a way of testing why a process that is ‘capable’ isn’t performing right	Testing at Intermediate stages
	Stream-to-stream testing
	Time-to-time analyses
Choosing best solutions for process improvement or product design	Rank ordering attributes in terms of importance
	Quality Function Deployment
Understanding current state/processes	Process mapping
	Flow diagrams
	A3’s
Instituting remedies	Plan, do, check, act

More recently, CQI has been encouraged for use in healthcare [8]. Healthcare systems often have specific staff (i.e., quality officers) devoted to leading quality improvement efforts [9]. CQI modules are integral in medical training [10] including core curriculums for residency and fellowship education. There are a number of peer reviewed journals dedicated to publishing papers about CQI in healthcare--either as a primary or secondary focus of the journal (http://www.ihi.org/education/IHIOpenSchool/resources/Pages/WhereToSubmitYourWritingQIFriendlyPeerReviewedJournals.aspx) [11]. In addition, a recent series of articles promoted use of quality improvement tools in nephrology practice [12–16].

Although there has been interest in applying CQI within healthcare, and numerous reports of CQI processes that have been applied in healthcare, there appears to be a paucity of published literature systematically evaluating CQI use and its impact within medical contexts [17]. Past evaluations have been performed to assess effectiveness of CQI in some chronic conditions, [18] but there is heterogeneity in what exactly has been evaluated and how evaluations have been carried out [19]. This leaves some question as to how to interpret current evidence on the effectiveness of specific CQI strategies.

Over two decades ago, CQI was introduced and promoted for end-stage nephrology care by CMS [20]. Subsequent improvements were reported both in care patterns and clinical indices within U.S. dialysis populations [21–24]. But there is not yet a description on the state of evidence-based quality improvement science in the most recent time periods since, and across the continuum of nephrologic care. What quality tools have been used and evaluated? Where and how have they been successfully applied? Without having the answers to these questions, we are left promoting and adopting methods without fully understanding exactly how CQI has been used, where successes have been seen and whether changes observed are due to CQI itself or other factors.

We conducted a systematic review of the published literature to address these areas. The objectives of this review were to assess the extent CQI has been utilized in nephrology, identify temporal and/or spatial trends of use, assess if outcomes have been associated with CQI and determine which areas of nephrologic care have been most amenable to improvement efforts.

## Methods

With the aid of a professional informationist, we conducted a systematic review of the literature regarding quality improvement in nephrology.

To adhere to established best practices, we aligned our review process with the “Preferred Reporting of Items for Systematic Reviews and Meta-analyses” (PRISMA) recommendations [25].

### Protocol and eligibility

We defined our review to include full-text articles or reports in English that utilized continuous quality improvement to identify or address clinical, cost, efficiency, safety, communication, or process issues within nephrology. The search time-period was January 1, 2004 through October 1, 2014.

We considered research to be relevant if the CQI activities had been applied to participants (e.g., patients, providers), environmental issues, systems or processes. Our definition of “CQI” included methods that use quality improvement tools to (a) understand a process or system of care, (b) generate theories for why problems exist, (c) dissect why process issues in care occur, (d) choose best solutions to problems, (e) identify or improve current flow, or (f) institute remedies to fix problems. To be included, articles had to contain a CQI activity applied within an area of nephrology, e.g.,; chronic kidney disease (CKD), acute kidney injury, end stage renal disease (ESRD) or renal transplantation. Case reports, clinical practice guidelines, “thought” pieces, editorials, “state-of-affairs-reports”, literature reviews, news reports and meta-analyses were excluded.

To assess the nature and quality of the CQI work cited, articles were evaluated for whether a predefined outcome was measured, whether there was a follow up measurement post CQI activity, and if so, whether improvement in an outcome was identified.

### Information sources and search strategy

Studies were identified by searching electronic databases and sources of gray literature. The literature search was applied to PubMed MEDLINE, Scopus, Web of Science, Cumulative Index of Nursing and Allied Health Literature (CINAHL), Google Scholar, and ProQuest Dissertation Abstracts. Pre-defined search algorithms were used to query PubMed MEDLINE as one part of the literature review and other databases as outlined above. Search terms used in PubMed MEDLINE were: ((("Nephrology" [Mesh] OR nephrology[tiab] OR renal[tiab] OR "Kidney Diseases" [Mesh]))) AND ((("Total Quality Management" [MeSH Terms] OR "Quality Improvement" [MeSH Terms] OR "quality improvement"[tiab] OR "Six Sigma" [tiab] OR "continuous quality improvement"[tiab] OR "total quality management" [tiab] OR "root cause" [tiab] OR "value stream" [tiab]))) NOT (Comment [ptyp] OR Editorial [ptyp] OR Letter [ptyp]). Terms were adapted for specific needs of each additional search engine.

### Review team

An expert panel was convened for the review. This panel included one member both with a doctorate in Instructional Technology and a Masters in Library and Information Sciences (NM), four practicing nephrologists who also had training in epidemiology and public health (MH, PR, JHS, JWN), and one member with a doctorate in psychology, who also oversees a patient safety and quality scholars program for our health system (FJS).

**Fig. 1:**
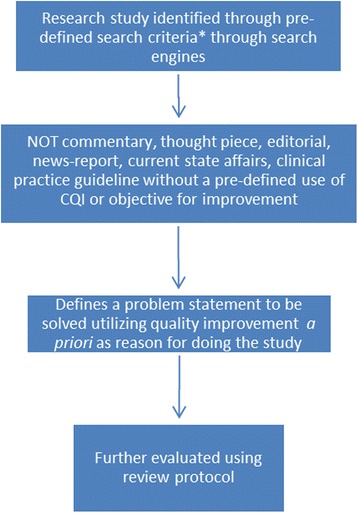
Algorithm for review. *Use of quality improvement with a pre-defined methodology to identify or address clinical, cost, efficiency, safety, communication or process issues within nephrology

Because definitions of CQI vary, several pre-review meetings of this team were used to define the scope of the review, define the questions we sought to answer and to establish a consensus definition of CQI. From these pre-review meetings we developed an initial algorithm for the review (Fig. 1). Two rounds of piloting the review process occurred whereby all members of the review panel reviewed an initial set of 20 citations, and subsequently met to discuss their independent reviews; the purpose was to clarify questions that arose and refine the review approach where needed. This served to maximize transparency, and ensure consistency in the reviews.

### Study Selection and data abstraction

Two nephrologist reviewers evaluated the eligibility of each citation independently using citation title and abstract. One reviewer reviewed all citations (JWN) and each citation was co-reviewed by an additional reviewer (MH, JHS, or PR). Full text articles were retrieved for any citation deemed potentially relevant. The reviewers then met to review their independent evaluations of inclusion with the final eligibility confirmed in this co-review. Disagreements on inclusion were resolved by consensus. Data abstraction occurred once a citation was deemed to have met the eligibility criteria and agreed upon by consensus for inclusion by the two independent reviewers.

The team developed a set of characteristics that would be used to define the nature of the CQI activity described in each eligible study. Pre-defined forms for data abstraction were used in assessing these studies. Fields assessed included whether a specific CQI tool was described and if so, which tool. We assessed which outcomes were measured, the type of outcome, and whether there was a comparison group or baseline measure that showed improvement. We assessed the study trial type including whether the study was randomized with a control group. Additional data included continent of origin, year published, primary journal discipline, and area of nephrology where CQI was applied.

### Data analysis and reporting

Abstracted data was summarized as frequency (n) and percent (%). Citations were managed using reference manager RefWorks (Copyright2015, Proquest LLC) and Endnote X4 (Copyright 1988-2010, Thompson-Reuters).

## Results

**Fig. 2:**
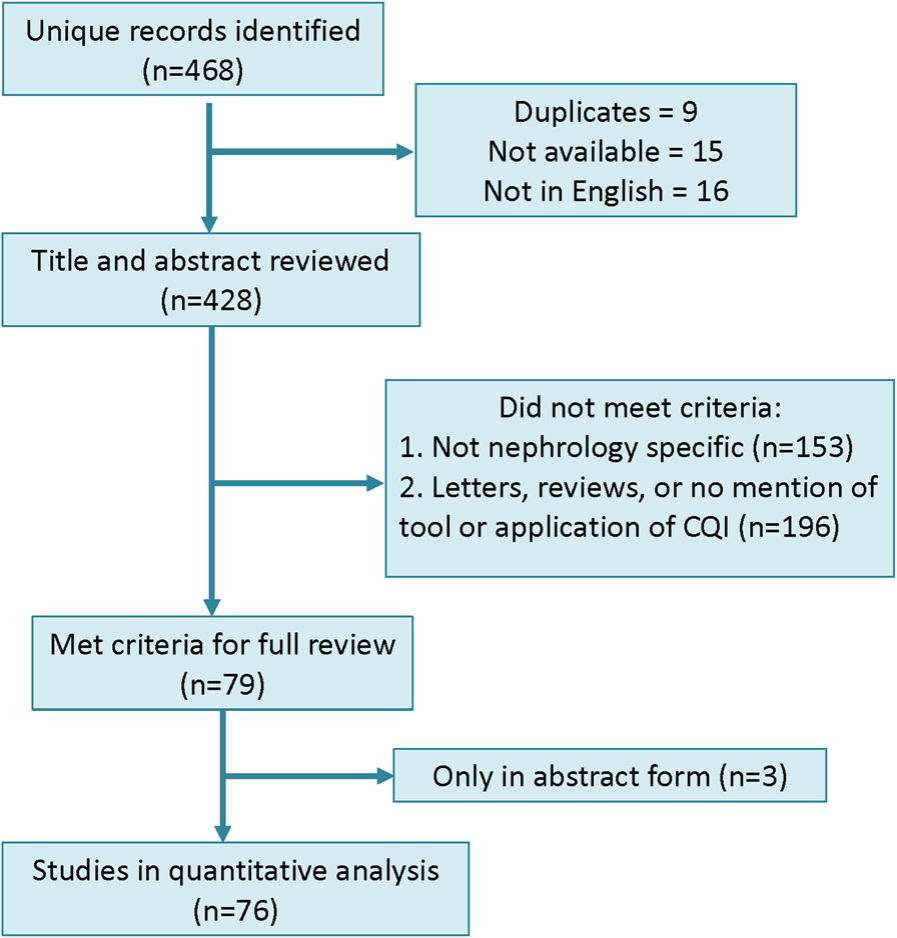
PRISMA flow chart of our review

Figure 2 shows the flow of information through the different phases of our review. The search identified 468 citations from January 1, 2004 through October 1, 2014. After adjusting for duplicates, those not available in English, and those with no abstract available in print or online, we reviewed 428 citations. Of these, 153 were not on a topic directly related to nephrology and 196 did not meet criteria for inclusion (e.g., letters to editor, reviews, no clear mention of any CQI as a purpose at study outset, or no mention of CQI at all in methods). Seventy-nine met criteria for full review; three were only available in abstract form. Seventy-six were included in final quantitative analyses (Fig. 2).

**Fig. 3:**
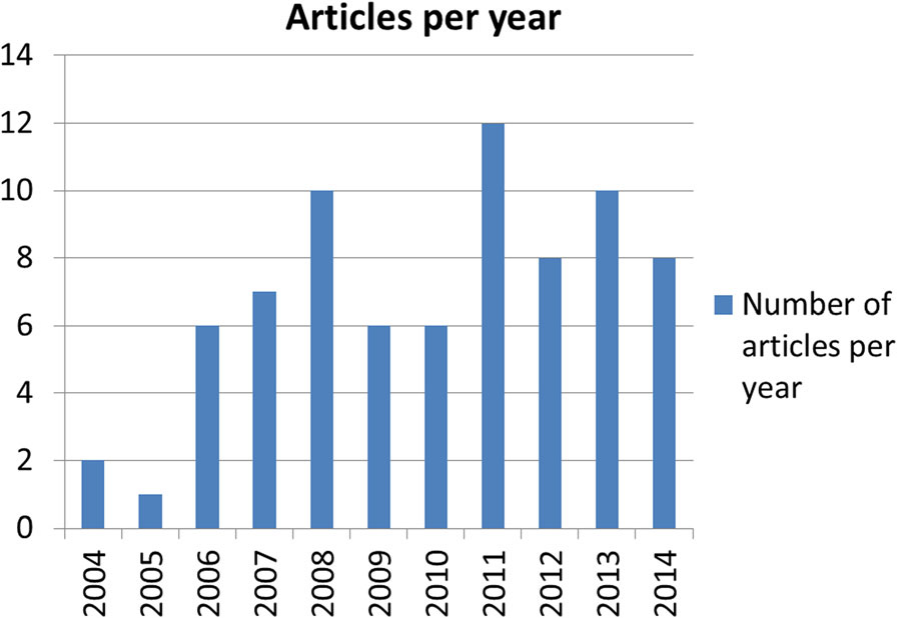
Number of articles published, by year

Characteristics of the included 76 studies are as follows. Fifty-six (74%) explicitly provided mention of a specific CQI tool in their methods (e.g., value stream mapping, plan-do-check-act, root cause analysis or multi-disciplinary teams). Sixty-eight (89%) were published in biomedical and allied health journals. The number of studies peaked in 2011, with 12 reported in the literature (Fig. 3). Forty studies (52%) originated in North America, followed by 23 (30%) from Europe, and a total of 5 from Asia, 5 from Australia, 2 from South America, and 1 from Africa.

**Fig. 4:**
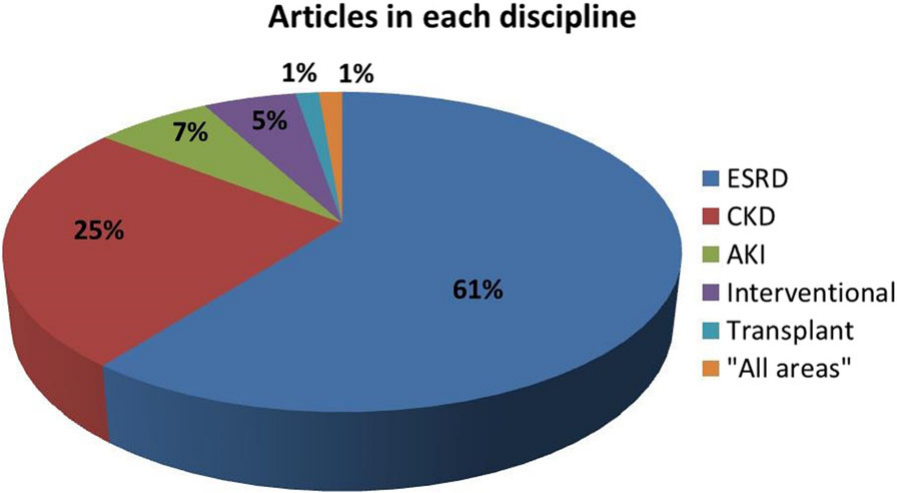
Breakdown of studies within each sub-discipline (note, some studies may have spanned > 1 discipline e.g., Interventional and ESRD, the primary discipline is shown)

The majority of studies focused on CQI use within ESRD (61%), although there was overlap across sub-disciplines within nephrology in nine of these. One study spanned improvement efforts applicable to all of nephrology. Figure 4 shows a breakdown of studies within each nephrology sub-discipline (Fig. 4).

Table 2 summarizes by discipline the types of outcomes measured, where CQI techniques were applied and whether interdisciplinary teams were involved. Most outcomes were clinical (e.g., 34 of the 46 studies in ESRD or 74%). Explicit CQI techniques were most often used to identify problems or causes to problems, rather than focused on solutions to address them (Table 2).

**Table 2:** Summary – outcomes measured and applications of CQI by primary discipline, n (%). [*N* = 76 total studies; references below table]

	ESRD (*n* = 46)	CKD (*n* = 19)	Transplant (*n* = 1)	AKI (*n* = 5)	Interventional (*n* = 4)	Other (*n* = 1)
Outcomes						
Baseline/one-time only	13 (28%)	6 (31%)		1 (20%)		1 (100%)
Pre/post outcome measures	29 (63%)	7 (37%)	1 (100%)	4 (80%)	3 (75%)	
Unclear or no outcomes measured	4 (9%)	5 (26%)			1 (25%)	
Type(s) of outcome(s)^a^						
Clinical	34 (74%)	9 (47%)	1 (100%)	3 (60%)	2 (50%)	
Cost/efficiency	14 (30%)	4 (21%)	1 (100%)	2 (40%)	1 (25%)	
Explicit CQI technique(s) used^b^						
To identify problems	33 (72%)	15 (79%)	1 (100%)	5 (100%)	2 (50%)	
To identify/address solutions	19 (41%)	7 (36%)	1 (100%)	4 (80%)	1 (25%)	1 (100%)
Use of interdisciplinary teams	28 (61%)	12 (63%)	1 (100%)	2 (40%)	3 (75%)	

^a^Note: studies may have included more than one type of outcome

^b^Note: studies may have used CQI to identify problems and address solutions

ESRD references: [27, 31, 45–88]

CKD references: [28, 89–106]

Transplant reference: [107]

AKI references: [26, 30, 108–110]

Interventional nephrology references: [111–114]

Other (this study was aimed at nephrology discipline in general) reference: [115]

In addition, four of the 76 studies were conducted within pediatric nephrology [26–29]. One used statistical process control to identify improvements in an intervention to increase CRRT filter life [26]. Another attempted to improve the pediatric dialysis experience using a ‘nursing intervention’- not well described [27]. Two other studies focused on populations in CKD not on dialysis-- developing protocols for managing nephrotic syndrome [29] and improving patient satisfaction [28].

Forty-two out of the 76 studies (55%) reported both a baseline or first-measured outcome and post measure outcome *along* with improvement. Of these, 23 focused on clinical outcomes, six were cost/efficiency related, and 11 included outcomes both clinical and cost/efficiency related. Following the trend of our overall sample, the majority of these studies (27 of the 42, 64%) occurred in end stage populations receiving hemodialysis—focusing on improving clinical indices (fistula rates/patency, anemia, high blood pressure). Some spanned systems of care; for example, one study used multi-disciplinary teams led by quality coaches to reduce contrast induced acute kidney injury across six hospitals [30].

Only one study of the 76 was randomized and controlled. It was not blinded due to the nature of the study [31]. Seventeen dialysis patients were randomized to a home blood pressure monitor plus an education intervention and 17 received usual care. Significant improvements in blood pressure were observed in the intervention group. The intervention was nursing driven and focused on home blood pressure monitoring to support patients in engaging in their own blood pressure management [31].

A table that lists all 76 articles and key areas of data we abstracted is located in the Additional file. (Additional file 1).

## Discussion

Despite calls for healthcare reform [8] and funding for research promoting novel methods to improve care, [32] our review revealed little rigorous research examining impact or efficacy of CQI in nephrology. We identified only 76 published studies in a 10 year span. The majority of studies (66% including ESRD and interventional-related studies) focused at end stages of renal disease, missing an early opportunity to use CQI to optimize processes to prevent or abate disease progression. When applied, many CQI efforts used multi-disciplinary teamwork, not taking advantage of other well-developed methods like statistical process control or plan-do-check-act. Only slightly more than half of the studies included pre- and post-intervention outcome measures. These findings highlight a significant need for increased rigor in CQI research and reporting in nephrology.

CQI methods have reportedly been applied to multi-million (even billion) dollar industries outside of healthcare with considerable success. Boeing, a leading U.S. aerospace corporation, reported a 54 % reduction in build hours and 218 % increase in its build rate of helicopters during the 1990’s [33]. They reduced defects 90% while saving 1.5 million labor hours and halving delivery time of aircraft. CQI in healthcare has shown promise as well, [34] highlighted in experiences at the Virginia Mason Medical Center [35]. By adapting and implementing the Toyota Production System of quality improvement into its hospital and clinics, [36] teams redesigned care to better meet patients’ needs, reduced inefficiencies in nursing, and increased direct nursing care activities [35]. Given these successes, it is unclear why CQI has not been studies or reported on more widely and rigorously within the nephrologic literature.

Medical literature ideally involves a high level of scrutiny prior to publication-including a peer-reviewed vetting process and assurances that studies are designed to examine whether an intervention impacts outcomes. Perhaps CQI research designs or interventions are not familiar enough to traditional journals or do not measure up to rigors established for reporting [37]. It may also be that applications of CQI in nephrology are intended to meet internal needs of practices and not thought of as applicable to publish. One example may be found in the ESRD networks: all networks engage in regular regional improvement activities and routinely set out to achieve important clinical goals using CQI toolkits, [38] but are not typically designed for research publication. Although the study of CQI implementation may not be thought of as traditional research in the past, its use is promoted in medical literature, including a very recent and lengthy series of nephrology-specific articles [12–16].

Taken together, this indicates there may be a contradiction between what we do and promote through research to achieve best outcomes and what we do and promote in “real-life” to actually realize them. CQI efforts in nephrology should be published so that we can learn from others about how these tools work and which ones provide the most benefits. This review serves as a starting point to better understand these areas. But as with prior evaluations in other aspects of healthcare point out, [17, 19] a challenge remains for future studies using CQI; not only to report on how CQI has been implemented, but to acknowledge and address when changes do occur, that they occur because of CQI attributes and not because of other factors.

Another reason for paucity of reporting on CQI efforts in research or other literature may be that tools within CQI have not fully caught on with practitioners or researchers in the nephrology community. As stated above, this may change because there are a growing number of recent publications promoting its use [39, 40]. Moreover, CMS has promoted a focus on quality and performance measurements (i.e., quality metrics) to evaluate care and practice in ESRD for some time [20, 41]. This foundational work in the 1990’s and early 2000’s related to evaluation of quality initiatives in ESRD populations [21–24] may be the reason for our observation of a preponderance of studies in dialysis populations more recently.

But CQI can provide more than a basis to evaluate metrics in a focused area of one medical discipline. CQI offers tools to address problems and optimize processes across disparate areas of disease management. It can provide a foundation for culture and paradigm changes that support and sustain improvements, across the continuum of kidney care, even prior to renal replacement (Table 3).

**Table 3:** Quality assurance vs continuous quality improvement adapted and reprinted from U.S. Department of Health and Human Services, HRSA, “What is the difference between quality improvement and quality assurance?” 3/18/15

	Quality assurance	Continuous quality improvement
Motivation	Measuring compliance with standards	Continuously improving processes to meet standards
Means	Inspection	Prevention
Attitude	Required, defensive	Chosen, proactive
Focus	Outliers: *"bad apples"* Individuals	Processes Systems
Scope	Medical provider	Patient care
Responsibility	Few	All

[http://www.hrsa.aquilentprojects.com/healthit/toolbox/HealthITAdoptiontoolbox/QualityImprovement/whatarediffbtwqinqa.html]

There are potential limitations of this review. As in all reviews, there is risk for incomplete retrieval of all studies on topic-in this case CQI within nephrology. We attempted to minimize this risk by using broad and inclusive search criteria with multiple terms that encompassed both CQI and nephrology, spanning over ten years. We also applied our search outside of usual sources and into the gray literature, recognizing that non-traditional distribution channels may be more amenable to reporting on CQI efforts. Another limitation is that there is not one recognized and singular definition for CQI nor for what is deemed “CQI research”. We attempted to address this in several meetings of our expert panel to unify our approach and definitions, clarify potential relevant search terms and create a review protocol which we pilot-tested and then revised for clarity prior to the full review.

There are several important implications of this review. Only 19 of the 76 studies focused on pre-dialysis CKD with only four occurring in patients where kidney disease strikes early (pediatrics). With the majority of studies focused on end stages, clearly there remains a need to shift an equal if not larger focus from research in tertiary prevention to primary and secondary prevention [42]. There may also be a need to expand current efforts to educate those within nephrology research on CQI tools and how they can be applied across the spectrum of disease, a process in its infancy. With the majority of publications arising from North America, there may be opportunity to promote CQI education not only in U.S. nephrology training, but across the globe. Although there are some metrics established related to ESRD performance, we need more than metrics to effect the changes necessary to improve overall health in our populous. Lastly, as non-traditional research efforts expand, areas of research like implementation science (“(the) study of methods to promote…systematic uptake of research findings and other evidence-based practices into routine practice” [43] may lead to more reporting of CQI related studies [44].

## Conclusions

To our knowledge this is the first systematic review of the published literature to determine how CQI has been applied to optimize outcomes in nephrology. We found that although there are CQI applications reported, the majority of studies mainly focused at end stages of disease, with comparison outcomes assessed just over half the time. There remains a significant and missed opportunity to examine CQI effectiveness and impact on outcomes, potentially as a way to usher in the sweeping improvement that is needed to address the gap between low quality and high cost seen in many chronic diseases including CKD.

## Additional file

Additional file 1: Final (Report) analysis file QI in CKD 10 year. (PDF 97 kb)

## Abbreviations

CINAHL: Cumulative Index of Nursing and Allied Health Literature
CKD: Chronic kidney disease
CQI: Continuous quality improvement
ESRD: End stage renal disease
PRISMA: Preferred reporting of items for systematic reviews and meta-analyses
