# Supplementary material for: Continuous quality improvement in nephrology: a systematic review
Source: BMC Nephrol. 2016 Nov 24;17:190. doi: 10.1186/s12882-016-0389-1 (PMC5121952; doi:10.1186/s12882-016-0389-1)
Supplement: Additional file 1: — Final (Report) analysis file QI in CKD 10 year. (PDF 97 kb) [file 12882_2016_389_MOESM1_ESM.pdf]

Year	Citation	Primary Discipline	Outcome post CQI or after baseline	First or one-time baseline measure	Outcome measure purported to show improvement	Measure/Outcome is clinical	Measure/Outcome is cost, efficiency or output related	Discuss or mention QI technique/process	Was a CQI method used to identify or explain problem	Was a CQI method used to generate a solution or solve a problem	Methods explicitly stated	Discussed or referenced use of multi or interdisciplinary team	Continent	Journal type	Additional Notes
2014	Arce, Jose M., Hernando, Luis, Ortiz, Alberto, et al. Designing a method to assess and improve the quality of healthcare in Nephrology by means of the Delphi technique 2014	all of nephrology	Yes	No	not applicable or unclear	no	no	Yes	not applicable	yes	Delphi technique	no	Europe	biomed	Used Delphi technique to gain consensus on nephrologist agreement on quality measures
2014	Brown, J. R., Solomon, R. J., Sarnak, M. J., et al. Reducing Contrast-Induced Acute Kidney Injury Using a Regional Multicenter Quality Improvement Intervention 2014	AKI	Yes	Yes	Yes	Yes	No	Yes	combination of tools	Yes	Multidisciplinary team, Benchmarking, Structure interviews and Quality improvement training	Yes	North America	biomed	Identified best practices through literature and benchmark sites, formed teams led by QI microsystem coaches to reduce AKI
2014	Lorch, Jonathan A., Pollak, Victor E. Continuous Quality Improvement in Daily Clinical Practice: A Proof of Concept Study 2014	ESRD	Yes	Yes	Yes	Yes	Yes	Yes	other	Yes	Multidisciplinary team	Yes	North America	biomed	Used retrospective data to design protocol and initiate multidisciplinary anemia management team use it to improve hematologic indices
2014	Majoni, S. W., Ellis, J. A., Hall, H., Abeyaratne, A. and Lawton, P. D. Inflammation, high ferritin, and erythropoietin resistance in indigenous maintenance hemodialysis patients from the Top End of Northern Australia 2014	ESRD	No	Yes	not applicable or unclear	Yes	No	No	No	No	Compared clinical data to established guidelines of practice	not applicable or unclear	Australia	biomed	Retrospective assessment of hematologic indices associated with anemia and determination of anemia management adequacy in population from Northern Australia
2014	Nicholas, J., Shaw, C., Pitcher, D. and Dawnay, A. UK renal registry 16th annual report: Chapter 12 biochemical variables amongst UK adult dialysis patients in 2012: National and centre-specific analyses 2014	ESRD	Yes	Yes	not applicable or unclear	Yes	No	No	No	No	Compared clinical data to established guidelines of practice, one time and longitudinally	No	Europe	biomed	Retrospective assessment of bone mineral indices and determination of adequacy of bone mineral management in UK
2014	Rafiq, M., McGovern, A., Jones, S., et al. Falls in the elderly were predicted opportunistically using a decision tree and systematically using a database-driven screening tool 2014	CKD	No	Yes	not applicable or unclear	Yes	No	Yes	rootcause	Yes	Decision tree taken as a form of root cause for problem analysis	No	Europe	biomed	As part of a QI in CKD study, determined causal factors for falls in elderly population with CKD and created decision support for clinicians to identify those at high risk
2014	Tahir, M., Hassan, S., De Lusignan, S., Shaheen, L., Chan, T. and Dmitrieva, O. Development of a questionnaire to evaluate practitioners' confidence and knowledge in primary care in managing chronic kidney disease 2014	CKD	No	Yes	not applicable or unclear	not applicable or unclear	not applicable or unclear	Yes	combination of tools	No	Multi-disp team, rank ordering topics to include in survey	Yes	Europe	biomed	As part of a QI in CKD study, used multi-disciplinary team to design survey to assess provider confidence managing aspects of CKD
2014	Wong, L. P., Yamamoto, K. T., Reddy, V., et al. Patient education and care for peritoneal dialysis catheter placement: a quality improvement study 2014	ESRD	No	Yes	not applicable or unclear	Yes	No	Yes	multi-interdisp team	not applicable or unclear	Multi-disp team	Yes	North America	biomed	As part of QI / fellow project, a multi-disp team developed and implemented patient survey to identify issues in PD
2013	Appleby, Sharon. SHARED CARE, HOME HAEMODIALYSIS AND THE EXPERT PATIENT 2013	ESRD	Yes	Yes	Yes	Yes	Yes	Yes	combination of tools	not applicable or unclear	Multi-disp team, process flow, education and testing	Yes	Europe	allied health	Introduced 'shared care' program to reduce wait times until home HD, increase efficiency, and patient self-management
2013	Bissonnette, J., Woodend, K., Davies, B., Stacey, D. and Knoll, G. A. Evaluation of a collaborative chronic care approach to improve outcomes in kidney transplant recipients 2013	Transplant	Yes	Yes	Yes	Yes	Yes	Yes	multi-interdisp team	Yes	Multi-disp team, redesign of care process, protocols & standardized care based on guidelines available	Yes	North America	biomed	Implemented a redesigned care delivery process with multi-disciplinary team and examined impact to clinical/effiency related outcomes
2013	Chen, L. L. A preliminary review of the medication management service conducted by pharmacists in haemodialysis patients of Singapore General Hospital 2013	ESRD	Yes	Yes	Yes	Yes	not applicable or unclear	Yes	multi-interdisp team	Yes	Multi-disp team, pilot redesign of care	Yes	Asia	allied health	Designed and implemented a pharmacist medication management system to reduce drug related problems in patients
2013	Chenoweth, Carolyn. Reducing nursing needlestick injuries in haemodialysis clinics: a quality improvement program 2013	ESRD	Yes	Yes	Yes	Yes	No	Yes	rootcause	Yes	Root cause analyses, pre/post CQI measurements, team review with education protocols	Yes	Australia	biomed	Identified causes to needlestick injuries and utiliized CQI methods to address causes and reduce injuries
2013	Esposito, P., Benedetto, A. D., Tinelli, C., et al. Clinical audit improves hypertension control in hemodialysis patients 2013	ESRD	Yes	Yes	Yes	Yes	No	Yes	multi-interdisp team	not applicable or unclear	Multi-disp team with audit process first involving education of physicians through peer group	Yes	Europe	biomed	Utilized peer groups and audit process with educational component to optimize BP in patients receiving HD

2013	Lewis, Samantha, White, Yvonne. Identification of unplanned activity in a regional home dialysis training unit 2013	ESRD	No	Yes	not applicable or unclear	No	Yes	No	none	No	Identification of inefficiencies deemed as unplanned nursing activities as part of quality improvement in home dialysis	No	Australia	allied health	Design and implementation of tracking system to identify amount of time spent on unplanned activites during two-week period in home dialysis
2013	Mottes, T., Owens, T., Niedner, M., Juno, J., Shanley, T. P. and Heung, M. Improving delivery of continuous renal replacement therapy: impact of a simulation-based educational intervention 2013	AKI	Yes	Yes	Yes	Yes	Yes	Yes	Serial / statistical process control	Yes	Statistical process control, team based and simulation for education	Yes	North America	biomed	Implemented a simulation education program to reduce down time and extend filter life in pediatric critical care CRRT
2013	Schachter, M. E., Romann, A., Djurdev, O., Levin, A. and Beaulieu, M. The British Columbia Nephrologists' Access Study (BCNAS) - a prospective, health services interventional study to develop waiting time benchmarks and reduce wait times for out-patient nephrology consultations 2013	CKD	Yes	Yes	Yes	No	Yes	Yes	combination of tools	Yes	Multi-disp teams, Delphi method to establish benchmarks, audits	Yes	North America	biomed	Used input from primary care and nephrology providers to establish benchmarks acceptable for referral to consult times, reduced wait times for consultations, most evidence in high risk patients
2013	Triamchaisri, S. K., Mawn, B. E. and Artsanthia, J. Development of a home-based palliative care model for people living with end-stage renal disease 2013	ESRD	Yes	Yes	not applicable or unclear	Yes	No	Yes	combination of tools	Yes	Multi-disp teams, and form of plan-do-check-act (although not formally stated)	Yes	Asia	allied health	Using input from patient and provider stakeholders, gained perspectives about issues facing patients with advanced CKD and developed intervention to assist/support palliative care
2013	Weale, A. R., study, team. The safer clinical systems project in renal care 2013	ESRD	No	No	not applicable or unclear	not applicable or unclear	not applicable or unclear	Yes	combination of tools	Yes	Process mapping, failure mode and effects analysis, hierachical task analysis, multi-disp teams	Yes	Europe	biomed	Describes initial phases of Safer Clinical Systems program to identify and eliminate potential safety breaches and optimize care for inpatients with kidney disease
2012	Ahya, S. N., Barsuk, J. H., Cohen, E. R., Tuazon, J., McGaghie, W. C. and Wayne, D. B. Clinical performance and skill retention after simulation-based education for nephrology fellows 2012	Interventional	Yes	Yes	Yes	not applicable or unclear	Yes	No	other	not applicable or unclear	Simulation education with pre/post education skills assessment	No	North America	biomed	Utilization of simulation education for skills building insertion of temporary dialysis catheters with pre and 2 post assessments
2012	Ball, L. K., Buss, J. A. Improving the fistula rate: the northwest renal network experience 2012	ESRD	Yes	Yes	Yes	Yes	No	Yes	Multi-interdisp team	Yes	Multi-disp teams, education program local and traveling to sites, follow up benchmarking shared with sites	Yes	North America	allied health	Multi-disp team created and led education sessions for HD centers re: need to increase AVFs - also included "coverletter" sent to sites relaying their metrics with improvements
2012	Dwyer, A., Shelton, P., Brier, M. and Aronoff, G. A vascular access coordinator improves the prevalent fistula rate 2012	Interventional	Yes	Yes	Yes	Yes	not applicable or unclear	na-not sure	none	Yes	Redesign of work process, protocols with process mapping, assume multi-disp team	Yes	North America	biomed	Established new work flow adding access coordinator and protocols / process maps to establish and improve AVF at HD site
2012	Elsayed, E., El-Soreety, W., Elawany, T. and Nasar, F. Effect of nursing intervention on the quality of life of children undergoing hemodialysis 2012	ESRD	Yes	Yes	Yes	Yes	No	No	none	not applicable or unclear	Used nursing intervention with education component	not applicable or unclear	Africa	allied health	Nursing education intervention administered to pediatric HD population improving domains of quality of life
2012	Hemmelgarn, B. R., Manns, B. J., Straus, S., et al. Knowledge translation for nephrologists: strategies for improving the identification of patients with proteinuria 2012	CKD	No	No	not applicable or unclear	not applicable or unclear	not applicable or unclear	Yes	multi-interdisp team	Yes	Multi-disp. Teams, form of root cause analyses with focus groups, concensus building nominal group technique	Yes	North America	biomed	Describes initial phases of knowledge-to-action plans for addressing low adherence to checking for proteinuria, at PCP and patient level
2012	Josland, Elizabeth, Brennan, Frank, Anastasious, Anastasia and Brown, Mark A. Developing and sustaining a renal supportive care service for people with end-stage kidney disease 2012	ESRD	No	Yes	not applicable or unclear	Yes	No	Yes	multi-interdisp team	not applicable or unclear	Multi-disp team delivering new mode of care	Yes	Australia	biomed	Describes initial phases of implementing new mode of "supportive" (palliative) care to patients with ESRD with goal to improve many clinical domains including quality of life
2012	Mukoro, F., Sweeney, G. and Mathews, B. Providing patients online access to their live test results: An evaluation of usage and usefulness 2012	CKD	No	Yes	not applicable or unclear	No	No	No	none	not applicable or unclear	Survey about online communication and results for patients	not applicable or unclear	Europe	other	Evaluated through a survey a prior implemented online system (Renal PatientView) that allows patients access to labs and online provider communication
2012	Parra, E., Arenas, M. D., Alonso, M., et al. Outcomes weighting for comprehensive haemodialysis centre assessment 2012	ESRD	No	No	not applicable or unclear	not applicable or unclear	not applicable or unclear	Yes	multi-interdisp team	Yes	Multi-disp team and weighting of quality indicators via focus groups	Yes	Europe	biomed	Established quality measures for HD using work group and multi-disp focus groups, and weighting to rank order in terms of importance

2011	Allen, A. S., Forman, J. P., Orav, E. J., Bates, D. W., Denker, B. M. and Sequist, T. D. Primary care management of chronic kidney disease 2011	CKD	No	Yes	not applicable or unclear	Yes	No	No	other	not applicable or unclear	Evaluation of quality of care via pre-defined criteria	No	North America	biomed	Evaluated 'quality' of CKD care assessing four domains including monitoring, CVS disease, bone mineral, drug safety
2011	Bayliss, E. A., Bhardwaja, B., Ross, C., Beck, A. and Lanese, D. M. Multidisciplinary team care may slow the rate of decline in renal function 2011	CKD	Yes	Yes	Yes	Yes	No	Yes	multi-interdisp team	not applicable or unclear	Redesign of work process of care delivery, multi-disp team in care	Yes	North America	biomed	Comparison of clinical paramaters in patients receiving multi-disciplinary team based care versus those receiving usual care
2011	Chaudhry, A., Feest, T. UK Renal Registry 13th Annual Report (December 2010): Chapter 14: enhancing access to UK Renal Registry data through innovative online data visualisations 2011	CKD	No	No	not applicable or unclear	not applicable or unclear	not applicable or unclear	Yes	combination of tools	not applicable or unclear	Statistical process control, online monitoring and accessing quality data	not applicable or unclear	Europe	biomed	Describes enhanced access to UK renal registry of data, including online reporting that is longitudinal with expanded visualization to end users
2011	Desrochers, J. F., Lemieux, J. P., Morin-Belanger, C., et al. Development and validation of the PAIR (Pharmacotherapy Assessment in Chronic Renal Disease) criteria to assess medication safety and use issues in patients with CKD 2011	CKD	Yes	Yes	Yes	Yes	not applicable or unclear	Yes	combination of tools	Yes	Multi-disciplinary teams, rank ordering priorities	Yes	North America	biomed	Describes development and impact of using a set of criteria to detect drug related problems, developed my a multi-disciplinary team, test piloted in patient group
2011	Farrell, Anita, Riley, Kay, Wheeler, Susan and McLean, Scott. Application of critical thinking diagnostics in the renal setting 2011	ESRD	No	Yes	not applicable or unclear	No	No	No	other	not applicable or unclear	Use of self-assessment compared to expert assessment for individual improvement	No	Europe	allied health	Nursing led assessment of staff critical thinking skills inspired by prior undesired clinical incident
2011	Garcia Garcia, M., Valenzuela Mujica, M. P., Martinez Ocana, J. C., et al. Results of a coordination and shared clinical information programme between primary care and nephrology 2011	CKD	Yes	Yes	Yes	No	Yes	Yes	multi-interdisp team	not applicable or unclear	Multi-disciplinary team and alignment of care processes with quality guidelines	Yes	Europe	biomed	PCP and Nephrologist collaboration to optimize hypertension in patients with CKD through better referral process and use of prioritization criteria for visits versus explanatory report for denied consults including suggestions for management
2011	Gardner, J., Walton, J. Striving to be heard and recognized: nurse solutions for improvement in the outpatient hemodialysis work environment 2011	ESRD	No	Yes	not applicable or unclear	No	No	No	none	No	Interviews + focus groups with reference to rank ordering priorities	No	North America	allied health	Interviews and focus groups of nurses to identify and rank perspectives regarding work environment
2011	Hauser, Naomi, Anderson, Donna, Stevenson, Judy A., Kirschbaum, Suzanne M., Wetzel, Peggy and Hannah, Karen. A QIO-renal network collaboration experience: addressing care transitions 2011	ESRD	Yes	Yes	Yes	not applicable or unclear	not applicable or unclear	Yes	multi-interdisp team	not applicable or unclear	Multi-disciplinary team	Yes	North America	allied health	Describes multi-disciplinary team brought together to improve communication between settings in which dialysis patients receive care, implemented communication form; results vaguely suggest staff acceptance using form
2011	Nicole, A. G., Tronchin, D. M. Indicators for evaluating the vascular access of users in hemodialysis 2011	Interventional	No	not applicable or unclear	not applicable or unclear	No	No	Yes	combination of tools	not applicable or unclear	Multi-disciplinary team, Donabedian assessment, Delphi technique	Yes	South America	other	Utilization of multi-disciplinary team and QI techniques to develop indicators for monitoring and prevention of HD access complications
2011	Quinan, P., Beder, A., Berall, M. J., Cuerden, M., Nesrallah, G. and Mendelssohn, D. C. A three-step approach to conversion of prevalent catheter-dependent hemodialysis patients to arteriovenous access 2011	Interventional	Yes	Yes	Yes	Yes	No	Yes	combination of tools	not applicable or unclear	Multi-discplinary team, checklist with education	Yes	North America	allied health	Used multi-disciplinary team as part of a QI effort to transition HD patients from catheter to AVF/AVG
2011	Remon Rodriguez, C., Quiros Ganga, P. L., Gonzalez-Outon, J., et al. Recovering activity and illusion: the nephrology day care unit 2011	ESRD	Yes	Yes	Yes	Yes	Yes	Yes	combination of tools	Yes	Multi-discipinary team, plan-do-check-act, cause and effect, process redesign	Yes	Europe	biomed	Identified issues in care of kidney disease patients, designed and implemented a new model of care (day care unit) to improve clinical management of patietns with kidney disease

2011	Sledge, R., Aebel-Groesch, K., McCool, M., et al. Part 2. The promise of symptom-targeted intervention to manage depression in dialysis patients: improving mood and quality of life outcomes 2011	ESRD	Yes	Yes	Yes	Yes	No	Yes	other	No	Training program with intevention tracking and communication meetings	not applicable or unclear	North America	allied health	Describes implementation of a symptom targeted intervention program, established within social work care and its impact on patient depressive related symptoms, per their social worker
2010	Bajo, M. A., Selgas, R., Remon, C., et al. Scientific-technical quality and ongoing quality improvement plan in peritoneal dialysis 2010	ESRD	No	No	not applicable or unclear	not applicable or unclear	not applicable or unclear	Yes	multi-interdisp team	No	Multi-disciplinary team, development of indicators to assess quality	not applicable or unclear	Europe	biomed	Describes development and outcomes of quality indicators for PD from a conscensus group of experts
2010	Heung, M., Adamowski, T., Segal, J. H. and Malani, P. N. A successful approach to fall prevention in an outpatient hemodialysis center 2010	ESRD	Yes	Yes	Yes	Yes	Yes	Yes	combination of tools	not applicable or unclear	Multi-disciplinary team, root cause analysis	Yes	North America	biomed	Identified reasons behind falls in patients at one HD site, implemented measures to reduce falls
2010	Khosla, N., Gordon, E., Nishi, L. and Ghossein, C. Impact of a chronic kidney disease clinic on preemptive kidney transplantation and transplant wait times 2010	CKD	Yes	No	Yes	Yes	No	Yes	multi-interdisp team	not applicable or unclear	Multi-disciplinary team, used in direct patient care	Yes	North America	biomed	Comparison of a multi-disciplinary care team versus usual care in patients with CKD, showing increased referral to renal transplantation in enhanced care
2010	Morton, A. R., Murphy, S., Hirsch, D., et al. Development and utility of a multi-dimensional grid to assess individual mineral metabolism control in hemodialysis patients: A potential aid for therapeutic decision making? 2010	ESRD	No	Yes	No	Yes	No	No	other	No	Assessment of patient bone mineral indices meeting established quality guidelines	not applicable or unclear	North America	biomed	As a first step of an initiative to improve management of mineral metabolism, this study describes achievement of patients within targets for established bone mineral guidelines
2010	Neyhart, C. D., McCoy, L., Rodegast, B., Gilet, C. A., Roberts, C. and Downes, K. A new nursing model for the care of patients with chronic kidney disease: the UNC Kidney Center Nephrology Nursing Initiative 2010	CKD	No	No	not applicable or unclear	not applicable or unclear	not applicable or unclear	Yes	combination of tools	not applicable or unclear	Multi-disciplinary team, root cause analysis, process mapping, process redesign suggested	Yes	North America	allied health	Describes initiative to redesign how nursing care is delivered, utilized teams and root cause analysis to identify problems and suggest a process redesign
2010	Stoves, J., Connolly, J., Cheung, C. K., et al. Electronic consultation as an alternative to hospital referral for patients with chronic kidney disease: a novel application for networked electronic health records to improve the accessibility and efficiency of healthcare 2010	CKD	Yes	Yes	Yes	No	Yes	Yes	combination of tools	not applicable or unclear	Multi-disciplinary team, process mapping, process redesign	Yes	Europe	allied health	Implementation of an e-consult service and comparison of referrals from GPs to Nephrologists, showing reduction in actual referrals and satisfaction with e-referrals compared to usual paper referrals
2009	Fontanesi, J., Mendoza, S., Bowers, D. and Reznik, V. Translating operational research to the medical community: using "guiding measurements" to improve the quality of healthcare delivery 2009	CKD	No	No	not applicable or unclear	not applicable or unclear	not applicable or unclear	Yes	combination of tools	Yes	Multi-disciplinary team, process mapping, process redesign, plan do check act	Yes	North America	other	Used combination of CQI methods to identify issues in care within pediatric population and compare nature of care to as is states, suggested process redesign to improve areas
2009	Hall, L., Gore, S. and Witten, B. Rehabilitation update: vocational rehabilitation: is your facility on track? 2009	ESRD	Yes	Yes	Yes	No	Yes	Yes	combination of tools	No	Multi-disciplinary team, benchmarking best practices and identifying problem practices	Yes	North America	allied health	Describes focused program to include and emphasize vocational rehabilitation in HD practices, resulted in improvement at target sites
2009	Lee, B. J., Forbes, K. The role of specialists in managing the health of populations with chronic illness: the example of chronic kidney disease 2009	CKD	Yes	Yes	Yes	Yes	Yes	na-not sure	none	Yes	Process redesign	not applicable or unclear	North America	biomed	Describes implementation by nephrology of change to referral process, whereby nephrologists solicited referrals through email from PCP's for patients deemed high risk for ESRD
2009	Lu, X. H., Su, C. Y., Sun, L. H., Chen, W. and Wang, T. Implementing continuous quality improvement process in potassium management in peritoneal dialysis patients 2009	ESRD	Yes	Yes	Yes	Yes	No	Yes	combination of tools	Yes	Multi-disciplinary team of care, education program	Yes	Asia	biomed	Utilized team based care to help patients receiving HD get serum potassium in acceptable ranges
2009	Ookalkar, A. D., Joshi, A. G. and Ookalkar, D. S. Quality improvement in haemodialysis process using FMEA 2009	ESRD	Yes	Yes	Yes	Yes	Yes	Yes	combination of tools	Yes	Multi-disciplinary team, Failure mode effects analysis	Yes	Asia	business	Utilized failure mode effects analysis and a cross functional team to identify and address risk factors for spread of viral infection (HCV) in HD
2009	Ridley, J., Wilson, B., Harwood, L. and Laschinger, H. K. Work environment, health outcomes and magnet hospital traits in the Canadian nephrology nursing scene 2009	ESRD	No	Yes	not applicable or unclear	Yes	Yes	No	none	not applicable or unclear	Used surveys to assess whether traits of magnet program aligned with nurse reported outcomes	No	North America	allied health	Used surveys to assess whether traits of magnet program aligned with nephrology nursing reported outcomes, including job satisfaction

2008	Alcazar, J. M., Arenas, M. D., Alvarez-Ude, F., et al. Preliminary results of the Spanish Society of Nephrology multicenter study of quality performance measures: hemodialysis outcomes can be improved 2008	ESRD	Yes	Yes	Yes	Yes	No	Yes	none	Yes	guidelines and recommendations assessement, computer generated reporting at intevals to local sites and peers	not applicable or unclear	Europe	biomed	Defined guidelines for clinical management and utilized computer capabilities to track HD site achievement, with communication to sites and inter-sites on achievement
2008	Arenas, M. D., Alvarez-Ude, F., Moledous, A., et al. Can we improve our results in hemodialysis? Setting quality objectives, feedback, and benchmarking 2008	ESRD	Yes	Yes	Yes	Yes	No	Yes	combination of tools	not applicable or unclear	Utilized benchmarking, assessment and reporting of site achievement of indicators	Yes	Europe	biomed	Utilized benchmark data and communication on achieving quality parameters at three HD sites, with improvement in clinical parameters at all sites at study end
2008	Bowe, D. IV iron therapy and anemia management in patients on hemodialysis: benefits of a revised CQI strategy 2008	ESRD	Yes	Yes	Yes	Yes	No	Yes	multi-interdisp team	not applicable or unclear	Defined updated protocol for clinical management	Yes	North America	allied health	Updated and implemented clinical protocol for anemia management at HD site
2008	Burg, G., da Silveira, D. D. Proposal of an environmental management model for nephrology services... World Congress of Nephrology Nursing, SÃ£o Paulo, April 22 to April 25, 2007 2008	ESRD	Yes	No	Yes	Yes	Yes	Yes	combination of tools	Yes	Multi-disciplinary input, process mapping	Yes	South America	biomed	Used patient, provider and housekeeping input and process mapping to address environmental improvements at HD site
2008	Patwardhan, M. B., Matchar, D. B., Samsa, G. P. and Haley, W. E. Opportunities for improving management of advanced chronic kidney disease 2008	CKD	No	Yes	not applicable or unclear	Yes	No	No	none	No	Chart abstraction to determine adherence to predefined quality metrics	No	North America	other	Describes results of chart abstraction on selection of patients in nephrology and primary care, comparing concordance of management/clinical indices with established
2008	Patwardhan, M. B., Matchar, D. B., Samsa, G. P. and Haley, W. E. Utility of the advanced chronic kidney disease patient management tools: case studies 2008	CKD	Yes	Yes	Yes	Yes	No	Yes	combination of tools	Yes	Multi-disciplinary teams and a 'toolkit' of resources	Yes	North America	other	Describes results of using a 'toolkit' with resources for providers to manage and educate patients on CKD
2008	Philipneri, M. D., Rocca Rey, L. A., Schnitzler, M. A., et al. Delivery patterns of recommended chronic kidney disease care in clinical practice: administrative claims-based analysis and systematic literature review 2008	CKD	No	Yes	not applicable or unclear	Yes	No	No	none	not applicable or unclear	Retrospective chart review comparing achievement of clinical parameters to guidelines	No	North America	biomed	To enhance current understanding of current CKD care, performed retrospective study describing care of patients with CKD
2008	Stamou, S. C., Camp, S. L., Reames, M. K., et al. Continuous quality improvement program and major morbidity after cardiac surgery 2008	AKI	Yes	Yes	Yes	Yes	No	Yes	other	not applicable or unclear	Mention CQI program, communication tools and protocol development emphasized	not applicable or unclear	North America	biomed	Describe reduction in sepsis and tamponade related events post CQI period in surgical services, included AKI as area of marginal improvement
2008	Williams, H. F., Fallone, S. CQI in the acute care setting: an opportunity to influence acute care practice 2008	AKI	No	Yes	not applicable or unclear	No	No	Yes	other	Yes	plan do check act	No	North America	allied health	Describes experiences of using CQI and plan do check act and generally positive impact in nursing / HD areas
2008	Wintz, R., Rosenthal, B. and Fadem, S. Z. The Physician Quality Reporting Initiative: a practical approach to implementing quality reporting 2008	CKD	No	No	not applicable or unclear	not applicable or unclear	not applicable or unclear	Yes	combination of tools	not applicable or unclear	Developed protocols, process maps and integrated into electronic system for reporting on quality indicators	Yes	North America	other	Describes development of electronic system including algorithms and protocols to assist site in reporting quality metrics / indices
2007	Ball, L. K., Treat, L., Riffle, V., Scherting, D. and Swift, L. A multi-center perspective of the Buttonhole Technique in the Pacific Northwest 2007	ESRD	Yes	Yes	Yes	Yes	Yes	Yes	none	Yes	Plan do check act, patient surveys	Yes	North America	allied health	Reports on multi-site success in implementing buttonhole technique for HD access, utilizing PDCA
2007	Harwood, L., Ridley, J., Lawrence-Murphy, J. A., et al. Nurses' perceptions of the impact of a renal nursing professional practice model on nursing outcomes, characteristics of practice environments and empowerment--Part I 2007	ESRD	Yes	Yes	Yes	No	No	No	other	Yes	Process redesign	No	North America	allied health	Describes nursing satisfaction and empowerment outcomes after implementation of a professional practice model
2007	Holtby, M. A. Know how it works before you fix it: a data analysis strategy from an inpatient nephrology patient-flow improvement project 2007	ESRD	No	Yes	not applicable or unclear	No	Yes	Yes	combination of tools	not applicable or unclear	Process mapping, multi-disciplinary team and value stream map	Yes	North America	allied health	Used team to identify existing patient flow in nephrology area and then excel to quantify time/date related to this flow as part of process improvement project

2007	Kauric-Klein, Z., Artinian, N. Improving blood pressure control in hypertensive hemodialysis patients 2007	ESRD	Yes	Yes	Yes	Yes	No	No	none	No	patient focused education and monitoring	No	North America	allied health	Randomized two groups of patients receiving HD to usual care versus home BP monitoring
2007	Li, Marilyn, Porter, Eveline, Lam, Robert and Jassal, Sarbjit V. Quality improvement through the introduction of interdisciplinary geriatric hemodialysis rehabilitation care 2007	ESRD	No	Yes	not applicable or unclear	Yes	Yes	Yes	none	Yes	multi-disciplinary team providing redesigned care	Yes	North America	biomed	Describes post implementation of a geriatric rehabilitation care program integrated with needs for HD
2007	Nguyen, V. D., Lawson, L., Ledeen, M., et al. Successful multidisciplinary interventions for arterio-venous fistula creation by the Pacific Northwest Renal Network 16 vascular access quality improvement program 2007	ESRD	Yes	Yes	Yes	Yes	No	Yes	combination of tools	Yes	multidisciplinary input using root cause analyses, multi-disp communication meetings	Yes	North America	biomed	Describes experience of a dialysis network using CQI techniques to improve AVF prevalence
2007	Tranter, S., Burns, T., Dobson, S., Graf, E., Ng, W. and Martinez, Y. Practice development in the hospital haemodialysis unit: improving calcium and phosphate management 2007	ESRD	na-not sure	Yes	not applicable or unclear	Yes	No	Yes	other	Yes	developed protocols, education and process flows	not applicable or unclear	North America	biomed	Utlized protocol development and algorithms, education to nursing staff to optimize bone mineral management in HD
2006	Chen, M., Deng, J. H., Zhou, F. D., Wang, M. and Wang, H. Y. Improving the management of anemia in hemodialysis patients by implementing the continuous quality improvement program 2006	ESRD	Yes	Yes	Yes	Yes	No	Yes	combination of tools	Yes	Find organize clarify uncover start, plan do check act, multi-disciplinary teams, process flow/mapping	Yes	Asia	biomed	Utilized several CQI tools to optimize anemia management
2006	Hinton, V., Fish, M. A care pathway for the end of life in a renal setting 2006	ESRD	Yes	Yes	Yes	Yes	No	Yes	other	Yes	Developed new care process, audit of charts to assess impact	not applicable or unclear	Europe	allied health	Used an integrated care plan, based on prior development of others, to optimize end of life care on renal wards
2006	Nicholas, P., Boys, J. and Best, J. Queensland collaborative for healthcare improvement: a model for the development of performance measures and quality improvement processes in renal dialysis 2006	ESRD	No	not applicable or unclear	not applicable or unclear	not applicable or unclear	not applicable or unclear	Yes	multi-interdisp team	not applicable or unclear	multi-disciplinary team	Yes	Australia	biomed	Describes a collaborative for healthcare improvement established specifically to develop performance measures and improve quality in renal dialysis
2006	Richardson, A., Reynolds, C. and Rodgers, R. Utilizing audit to evaluate improvements in continuous veno-venous haemofiltration practices in intensive therapy unit 2006	AKI	Yes	Yes	Yes	No	Yes	Yes	other	Yes	Used pre and post audits, education, identified change agents	not applicable or unclear	Europe	allied health	Describe a process of auditing, making improvements and re-assessing nursing practices in CVVHF within an ICU setting
2006	Tranter, S., Martinez, Y. and Rayment, G. A nurse-initiated iron management protocol for patients on hospital haemodialysis 2006	ESRD	Yes	Yes	Yes	Yes	Yes	Yes	multi-interdisp team	not applicable or unclear	multi-disciplinary team, revision of protocols	Yes	Europe	allied health	Instituted nurse intiated anemia management protocol in HD
2006	Wijnen, E., Planken, N., Keuter, X., et al. Impact of a quality improvement programme based on vascular access flow monitoring on costs, access occlusion and access failure 2006	ESRD	Yes	Yes	Yes	Yes	Yes	na-not sure	none	not applicable or unclear	instituted auditing/monitoring program	No	Europe	biomed	Describes costs and outcomes related to HD access before and after instituting access flow monitoring periodically as part of a quality improvement program

2005	Tigert, J., Chaloner, N., Scarr, B. and Webster, K. Development of a pamphlet: introducing advance directives to hemodialysis patients and their families 2005	ESRD	Yes	No	not applicable or unclear	No	No	Yes	multi-interdisp team	not applicable or unclear	multi-disciplinary team	Yes	Europe	allied health	Describes development of educational pamphlet regarding advanced care planning for patients receiving HD
2004	Sekkarie, M. Increasing the placement of native veins arteriovenous fistulae--the role of access surgeons' education and profiling 2004	ESRD	Yes	Yes	Yes	Yes	No	No	none	No	feedback / individual education	No	North America	biomed	Describes nephrologist experience of increasing fistula placements after instituting monitoring and feedback to access surgeons
2004	Stoffel, M. P., Barth, C., Lauterbach, K. W. and Baldamus, C. A. Evidence-based medical quality management in dialysis--Part I: Routine implementation of QiN, a German quality management system 2004	ESRD	Yes	Yes	Yes	Yes	No	No	none	not applicable or unclear	feedback / monitoring and reporting	not applicable or unclear	Europe	engineer	Describes improvements in HD clinical and management indices as a result of structured and specific feedback to sites on quality indicators
